# Peripheral Sensory Neurons Expressing Melanopsin Respond to Light

**DOI:** 10.3389/fncir.2016.00060

**Published:** 2016-08-10

**Authors:** Anna Matynia, Eileen Nguyen, Xiaoping Sun, Frank W. Blixt, Sachin Parikh, Jason Kessler, Luis Pérez de Sevilla Müller, Samer Habib, Paul Kim, Zhe Z. Wang, Allen Rodriguez, Andrew Charles, Steven Nusinowitz, Lars Edvinsson, Steven Barnes, Nicholas C. Brecha, Michael B. Gorin

**Affiliations:** ^1^Department of Ophthalmology, Jules Stein Eye Institute, David Geffen School of Medicine, UCLALos Angeles, CA, USA; ^2^Brain Research Institute, UCLALos Angeles, CA, USA; ^3^Department of Neurobiology and Medicine, David Geffen School of Medicine, UCLALos Angeles, CA, USA; ^4^Division of Experimental Vascular Research, Department of Clinical Sciences, Lund UniversityLund, Sweden; ^5^Department of Neurology, David Geffen School of Medicine, UCLALos Angeles, CA, USA; ^6^Departments of Physiology & Biophysics and Ophthalmology and Visual Sciences, Dalhousie UniversityHalifax, NS, Canada; ^7^Veterans Administration Greater Los Angeles Health SystemLos Angeles, CA, USA

**Keywords:** ipRGC, sensory ganglion, migraine, optic nerve injury, cornea, choroid

## Abstract

The ability of light to cause pain is paradoxical. The retina detects light but is devoid of nociceptors while the trigeminal sensory ganglia (TG) contain nociceptors but not photoreceptors. Melanopsin-expressing intrinsically photosensitive retinal ganglion cells (ipRGCs) are thought to mediate light-induced pain but recent evidence raises the possibility of an alternative light responsive pathway independent of the retina and optic nerve. Here, we show that melanopsin is expressed in both human and mouse TG neurons. In mice, they represent 3% of small TG neurons that are preferentially localized in the ophthalmic branch of the trigeminal nerve and are likely nociceptive C fibers and high-threshold mechanoreceptor Aδ fibers based on a strong size-function association. These isolated neurons respond to blue light stimuli with a delayed onset and sustained firing, similar to the melanopsin-dependent intrinsic photosensitivity observed in ipRGCs. Mice with severe bilateral optic nerve crush exhibit no light-induced responses including behavioral light aversion until treated with nitroglycerin, an inducer of migraine in people and migraine-like symptoms in mice. With nitroglycerin, these same mice with optic nerve crush exhibit significant light aversion. Furthermore, this retained light aversion remains dependent on melanopsin-expressing neurons. Our results demonstrate a novel light-responsive neural function independent of the optic nerve that may originate in the peripheral nervous system to provide the first direct mechanism for an alternative light detection pathway that influences motivated behavior.

## Introduction

The mammalian visual system uses distinct photoreceptors for image- and non-imaging-forming vision that send coded information to the brain via the optic nerve. By contrast, nociceptive, tactile, and proprioceptive information is encoded by the sensory neurons of the TG and dorsal root ganglia in the peripheral nervous system. Physically and functionally distinct, an association between photoreception and nociception is evidenced in photoallodynia, a clinical condition in which pain is caused or enhanced by dim or normal light (Digre and Brennan, 2012). The neural circuits for this association remain largely unknown (Noseda et al., 2010).

Rods and cones are classic photoreceptors that underlie night vision and high acuity, color vision respectively. The third class of retinal photoreceptors, intrinsically photosensitive retinal ganglion cells (ipRGCs), express melanopsin, which actively detects photons to directly signal multiple brain regions involved in circadian photoentrainment, pupillary light reflex (PLR), or contrast detection (Schmidt et al., 2011, 2014). Melanopsin has also been identified in rare cell bodies in the iris, in projections to the ciliary body and as a modulator of vascular development and dilation (Xue et al., 2011; Rao et al., 2013; Semo et al., 2014; Sikka et al., 2014). In mice, ipRGCs mediate innate and corneal surface damage-induced light aversion (Johnson et al., 2010; Semo et al., 2010; Thompson et al., 2010; Matynia et al., 2012, 2015) and in patients, are important for migraine-associated photoallodynia (Noseda et al., 2010). Although pain can be felt in various ocular tissues, the retina itself does not sense pain.

The sensory neurons of the TG and dorsal root ganglia detect both nociceptive and non-nociceptive stimuli. Nociceptors consist of small C fibers and medium-sized Aδ fibers that detect painful stimuli for chemical, thermal, and intense pressure stimuli (Le Pichon and Chesler, 2014; Palkar et al., 2015). While light can cause ocular and headache pain, the mechanisms underlying this association are thought to require higher order processing in the brainstem, with thalamic and somatosensory cortices for photoallodynia from migraine, or a direct contribution from corneal trigeminal nerves in photoallodynia from corneal surface damage (Moulton et al., 2009; Noseda et al., 2010; Rosenthal and Borsook, 2016). Hints have appeared in the literature, however, that suggest the trigeminal system may respond directly to light. Ablation of melanopsin-expressing neurons reduces corneal mechanical sensitivity and light aversion, suggesting that melanopsin influences the function of both Aδ and C fibers, respectively (Matynia et al., 2015). Furthermore, trigeminal blink reflexes are retained after optic nerve transection (Dolgonos et al., 2011) and bright light induces markers of neuronal activity in the trigeminal nucleus caudalis in rats (Okamoto et al., 2009, 2010).

The current study sought to determine if TG neurons could be directly influenced by light. We hypothesized that melanopsin, the photopigment mediating nocifensive behaviors such as the PLR, may also be expressed in small C fiber and Aδ fiber neurons. To have functional relevance, these neurons would show light-induced *ex vivo* electrophysiological responses and contribute to light-induced behavior independently of the optic nerve.

## Methods

### Mice

All experiments were performed in accordance with the institutional guidelines of the University of California at Los Angeles and the ARVO Statement for the Use of Animals in Ophthalmic and Vision Research. Wild type C57Bl/6J mice, Jackson Laboratories (Bar Harbor, MN), PDE6β^rd1/rd1^ mutant mice, generously provided by Dr. Deborah Farber (Bowes et al., 1990), and OPN4^EGFP^ mice (MRRC #033064-UCD) were used. OPN4^dta^ mice, a generous gift of Dr. Samer Hattar, in the C57Bl/6J genetic background were bred from heterozygous × heterozygous mating. ipRGC ablation was verified using a qualitative PLR assay (Matynia et al., 2012). All animals were tested between 3 and 12 months old. Both sexes were used for all experiments unless otherwise noted, however, group sizes were not appropriate to assess differences based on sex. All mice were group-housed, maintained in a 12:12 light/dark cycle with food and water *ad libitum*.

### Pharmacological agents

Atropine-sulfate (1%, Bausch & Lomb Inc., Tampa, FL) was applied bilaterally to corneal surfaces. Nitroglycerin (5 mg/mL, American Regent, Inc., Shirley, NY) was injected intraperitoneal at 10 mg/kg. Carprofen (50 mg/mL, Pfizer, Inc.) was injected subcutaneously at 5 mg/kg. Inhalant isoflurane (Phoenix), topical proparacaine hydrochloride (0.5%, Akorn, Inc.), ketamine (100 mg/mL, Phoenix) at 120 mg/kg, and xylazine (20 mg/mL, Lloyd Laboratories) at 10 mg/kg were used in anesthesia.

### Mouse tissue samples

Mice were transcardially perfused with a solution of 2% formaldehyde, 2.5% glutaraldehyde in 0.1 M Sodium Phosphate buffer, the optic nerve with eye cup removed, post-fixed in 1% Osmium Tetroxide in 0.1 M Sodium Phosphate buffer, dehydrated in ethanol, embedded in a resin containing a mixture of Epon 812 and Araldite, sectioned at 1 μm and stained with toluidine blue. Samples were visualized on a light microscope under a 40x oil objective (Axioplan, Carl Zeiss Meditec, Dublin, Ca) and images captured by a digital camera (CoolSNAP; Roper Scientific, Duluth, Ga). TGs were removed and fixed briefly in 4% PFA in 0.1 M Sodium Phosphate buffer. Samples were stepwise transferred into 10, 20, and 30% sucrose, embedded in OCT and 20 μm cryosections were mounted directly. Sections were washed in 1x PBS, stained with Hoechst 33342 (Sigma) then mounted in Vectamount with anti-fade (Vector Laboratories Inc., Burlingame), sealed, and imaged.

### Human tissue samples

TG were obtained at autopsy from adult subjects in accordance with the University Medical School (Lund) guidelines for ethics in human tissue experiments and were approved by the Regional Ethics Committee (case no LU-818-01) in accordance with the principles outlined in the Declaration of Helsinki. TG were removed from four subjects (three female; one male) with an average age of 78 years (67–89 years). None of the subjects suffered from any central nervous system disease and the cause of death was related to heart failure, septicemia or cancer. The tissue was collected within 24 to 36 h after death. Samples were immersed overnight in fixative consisting of 2% paraformaldehyde (PFA) and 0.2% picric acid in 0.1 mol/l phosphate buffer, pH 7.2. After fixation, the specimens were rinsed in sucrose-enriched (10%) Tyrode solution overnight, frozen and stored at −80°C. When the study was to start the ganglia were embedded in Tissue-Tek (Sakura Finetek, Europe), sagitally cryosectioned (10 μm) and the sections stored at −20°C until use.

### Immunohistochemistry

Ten micrometer thick cryosections of the four human TGs were washed in PBS with 0.25% Triton (PBS-T) for 15 min. Next, a melanopsin specific primary antibody was applied to the sections in a 1:1000 dilution in PBS+1% bovine albumin serum (BSA). The C-terminal anti-melanopsin antibody (rabbit #5J68) specific to human and macaque melanopsin, was previously characterized using preabsorption with the immunization antigen that abolished all staining (Hannibal et al., 2004, 2014; Dacey et al., 2005). The sections were incubated overnight in a damp incubation chamber at +8°C. The following day, sections were washed in PBS-T 2 × 15 min prior to the application of secondary anti-rabbit FITC-conjugated antibody (1:100, goat anti-rabbit, Cayman Chemical, Ann Arbor, MI). Slides were incubated at room temperature for 1 h, then washed three times in PBS-T for 15 min each followed by application of Vectashield mounting medium containing DAPI (Vector Laboratories, Burlingame CA, USA).

Double immunostaining was performed sequentially with melanopsin and CGRP (1:100, ab81887, Abcam UK; Eftekhari et al., 2015), as well as melanopsin and PACAP (1:100 sc166180, Santa Cruz Biotechnology, Santa Cruz, Ca), according to the previously described protocol. However, after the first secondary antibody was applied to first primary antibody, the slides were washed 2 × 15 min in PBS-T and the next primary antibody was applied to the sections. The sections were incubated yet again in +8°C overnight and the next day the appropriate secondary antibody (1:100, rabbit anti-mouse Alexa 594, Invitrogen, Carlsbad, CA) and mounting medium were applied as described above.

A negative control was included alongside each immunohistochemical staining in which the primary antibody was omitted to evaluate the specificity of the secondary antibody. The immunostaining was also confirmed using a different secondary antibody (donkey anti-rabbit, Alexa 594, Jackson Immuno Research, West Grove, PA) that resulted in an identical immunoreactivity. Furthermore, each experiment was repeated a minimum of three times to confirm reproducibility and specificity.

### Microscopy and cell counting

Human sections were examined using an epifluorescence microscope (Nikon 80i, Tokyo, Japan) combined with a Nikon DS-2MV camera. Areas of interest were photographed with 10x, 20x, or 40x lenses. The images were then processed using Adobe Photoshop CS3 (v10.0 Adobe Systems, Mountain View, CA) and images taken with different wavelength filters were superimposed over each other to determine any potential co-localization.

Mouse TG sections approximately every 100 μm were evaluated with a Zeiss laser scanning microscope 510 Meta (Zeiss LSM 710, Carl Zeiss, Thornwood, NY) with a Zeiss C-Apochromat x40 1.2 NA corrected water objective at a resolution of 1024 × 1024. Images are presented in figures either as a single image scan or a projection of 3–7 image scans (*z*-axis between 0.3 and 0.5 μm). The intensity levels and contrast of the final images were adjusted identically in Adobe Photoshop CS2 (v.9.02 Adobe Systems, Mountain View, CA). Morphometric analysis was performed using Image J. A total of 13191 mouse neurons were counted, of which 424 EGFP positive neuron somata diameters were measured.

### mRNA analysis

Total RNA was isolated from freshly dissected tissue using the RNeasy kit (Qiagen) and quantified on a Nanodrop UV spectrophotometer. 300–500 ng of total RNA was converted to cDNA using Multiscribe reverse transcriptase (High Capacity cDNA Reverse Transcription Kit, Applied Biosystems, Inc.). PCR was performed using two primer sets, forward melanopsin primer: 5′-CTGGGCTCCCTACTCCACT with reverse melanopsin primer: 5′-CGTCAGGATGTGCGAGTATC and forward actin primer: ctaaggccaaccgtgaaaag with actin primer: 5′-accagaggcatacagggaca. Amplification products were analyzed on a Qiaxcel capillary electrophoresis system (Qiagen).

### TG dissociation

Trigeminal ganglion neurons from mice were dissociated as previously described (Matsuka et al., 2007). Briefly, TG were harvested, chopped into small pieces and digested in 1 mg/mL Collagenase Type I (Worthington Biochemicals Corp) for 10 min at 37°C. Trypsin (Sigma) was added to 1 mg/mL final concentration and incubated for an additional 10 min at 37°C. Samples were centrifuged, washed twice in modified Hanks Buffered Saline Solution (–Mg, –Ca, Hyclone, Thermofisher), triturated, and resuspended in Neurobasal A (Gibco, Thermofisher) with B27 Supplement (Gibco, Thermofisher) and Glutamine (Gibco, Thermofisher). Cells were plated on poly-D-Lysine and laminin coated coverslips, and incubated at 37°C for a minimum of 2 h in the dark.

### Electrophysiology and calcium imaging

Whole cell patch clamp recording was performed on isolated trigeminal neurons perfused with extracellular solution containing (mM): 120 NaCl, 3 KCl, 2 CaCl_2_, 1.2 NaH_2_PO_4_, 10 glucose, 25 NaHCO_3_, pH7.4, bubbled with 95% O_2_/5% CO_2_. Whole cell patch recordings were performed at 21–23°C with fire-polished 8–12 MΩ resistance borosilicate patch pipets filled with internal solution (mM): 20 KCl, 120 K-gluconate, 0.3 GTP, 0.2 EGTA, 10 HEPES, 4 ATP-Mg, pH7.2, visualized under infrared light as described for ipRGCs (Graham et al., 2008; Do et al., 2009; Schmidt and Kofuji, 2009). A Multiclamp 700A (Molecular Devices, Sunnyvale, CA) running pClamp10 was used for current- and voltage-clamp recording, with calibrated light stimuli delivered via the objective from a shutter-controlled mercury lamp with neutral density and band-pass chromatic filters that produced irradiance measured in the recording chamber of 6.8 × 10^8^ photons/s/μm^2^ at 480 nm.

Calcium imaging was performed on trigeminal neurons isolated as above that had been loaded with 10 μM/mL fluo-4 AM (Invitrogen, Carlsbad, CA) for 60 min at 18°C. Neurons were observed with a long working distance 40x water immersion objective on a Nikon microscope equipped for epifluoresence imaging using a B2E filter set (stimulation at 450–490 nm, dichroic mirror at 505 nm and emission at 520–560 nm) and recorded with a camera. Bathing media and other experimental solutions were applied via a gravity flow system with a flow rate of 1.5 ml/min, and excess fluid was removed by suction. Images were obtained every 1 s at a resolution of 256 × 256. Fluorescence values are expressed in arbitrary units (AU).

### Behavioral assay

The light-aversion assay was performed as previously described with modifications (Matynia et al., 2012, 2015). Briefly, a two-chamber box with an open, light, and closed, dark section was used to measure time spent in the light compartment. An overhead LED lighting system, with adjustable illumination from 0 to 2000 Lux calibrated with a lightmeter (HHLM-2, Omega Engineering Inc.), a standard LED spectrum and diffusers provided uniform illumination in the open, lit side of the chamber. Behavior was monitored using an infrared light source and video camera with white light filter, and automated tracking and analysis were performed with Video Tracker (Med Associates, St. Albans, VT) and Activity Monitor (Med Associates, St. Albans, VT), respectively. Mice were acclimated to a dimly lit room (less that 10 Lux) for at least 45 min and dark-adapted for 10 min prior to testing. Light aversion was tested at 0 and 1000 Lux: the 0 Lux test was used as baseline to calculate aversion indices (AI).

### Optic nerve crush

Optic nerve crush was performed as previously described (Perez de Sevilla Muller et al., 2014; Rodriguez et al., 2014). Briefly, mice were anesthetized with isoflurane, the ocular surface anesthetized with proparacaine, a small incision made in the temporal conjunctiva, and the optic nerve exposed by gentle manipulation of surrounding tissue. Cross-action forceps were used to apply a 15 s crush ~0.5 mm behind the globe. Forceps were removed, antibiotic ointment applied to the incision site, carprofen administered for pain management and animals allowed to recover.

### Electroretinography and visually evoked potentials

Electroretinographic recordings (ERGs) and visually evoked potentials (VEPs) were performed as previously described (Nusinowitz et al., 2007). Briefly, mice were dark-adapted overnight, anesthetized and their eyes dilated. A gold wire electrode referenced to a similar gold electrode in the mouth, and a tail ground electrode, were used to record the ERG. For VEP recordings, a needle electrode was positioned subcutaneously 3 mm lateral to lambda over the contralateral visual cortex. The mouse head was positioned orthogonal to a large opening in a highly reflective Ganzfeld dome in which brief light flashes were presented using a Grass Photic Stimulator (PS33 Plus, Grass Instruments, Quincy, MA). Responses were amplified (CP511 AC amplifier, Grass Instruments), digitized (PCI-1200, National Instruments, Austin, TX) and computer averaged as described (Nusinowitz et al., 2007). ERGs were recorded under dark- and light-adapted conditions to evaluate rod- and cone-mediated retinal function, respectively. ERGs were recorded to achromatic flashes over a 4-log unit range of intensities (0.003–5.0 cd-s/m^2^). Cone-mediated ERG responses were obtained on a rod-saturating background (32.0 cd-s/m^2^). VEPs were recorded to the highest flash intensities (0.29–5.0 cd-s/m2) under dark-adapted conditions, which provided the most robust VEP signals to evaluate visual pathway function (Nusinowitz et al., 2007).

### Spectral domain optical coherence tomography

Ultra high resolution sd-OCT imaging was performed using a commercially available sd-OCT system (Bioptigen, Research Triangle Park, NC) as previously described (Engelhardt et al., 2012). Briefly, mice were anesthetized and pupils dilated as above. Recordings were made with a 50° field of view, yielding an image 1.5 mm in diameter centered on the optic nerve head. *En face* view C-scans were recorded, each consisting of 100 two-dimensional B-scans. Automated retinal thickness measurements were made using the analysis software (InVivoVue Diver 2.4) provided by the manufacturer. Up to seven retinal layers could be reliably segmented to provide volumetric data for the layer and region of interest. User driven retinal layer boundary marking tools were used to correct boundary lines as needed for each recorded B-scan. For all thickness measures, the optic nerve head component was excluded from analysis. Thickness measures were obtained for the total retina, the ganglion cell layer, and the outer retina.

### Von frey fiber assay

Corneal mechanical sensitivity was assessed using von Frey fibers by masked observers (Acosta et al., 2014). The central cornea of immobilized unanesthetized mice was touched with the fiber to the point of bending, held for 1 s and response graded as 0 = no blink, 1 = blink with reduced amplitude or speed, and 2 = normal blink. Three von Frey fibers in sequential order of increasing size were tested with five repetitions per fiber. Fiber sizes used were (diameter in mm, force in “g” in parentheses): 0.064 (0.008), 0.072 (0.020), 1.02 (0.040).

### Data and statistical analysis

AI were calculated for each mouse from the “time in light” as follows: (Time in Light_baseline_ – Time in Light_test_)/Time in Light_baseline_, with 0 Lux used as baseline. An AI of “0” represents no aversion and “+1” complete aversion. Graph Pad Prism (v5.0f, 2012) was used for statistical analyses for two-way ANOVA and *t*-tests as indicated. Significance is reported for two-tailed *t*-tests and Bonferroni correction applied for multiple comparisons as stated. All error bars represent standard error of the mean (SEM) values.

## Results

### Melanopsin is expressed in trigeminal ganglion neurons

**Figure 1:**
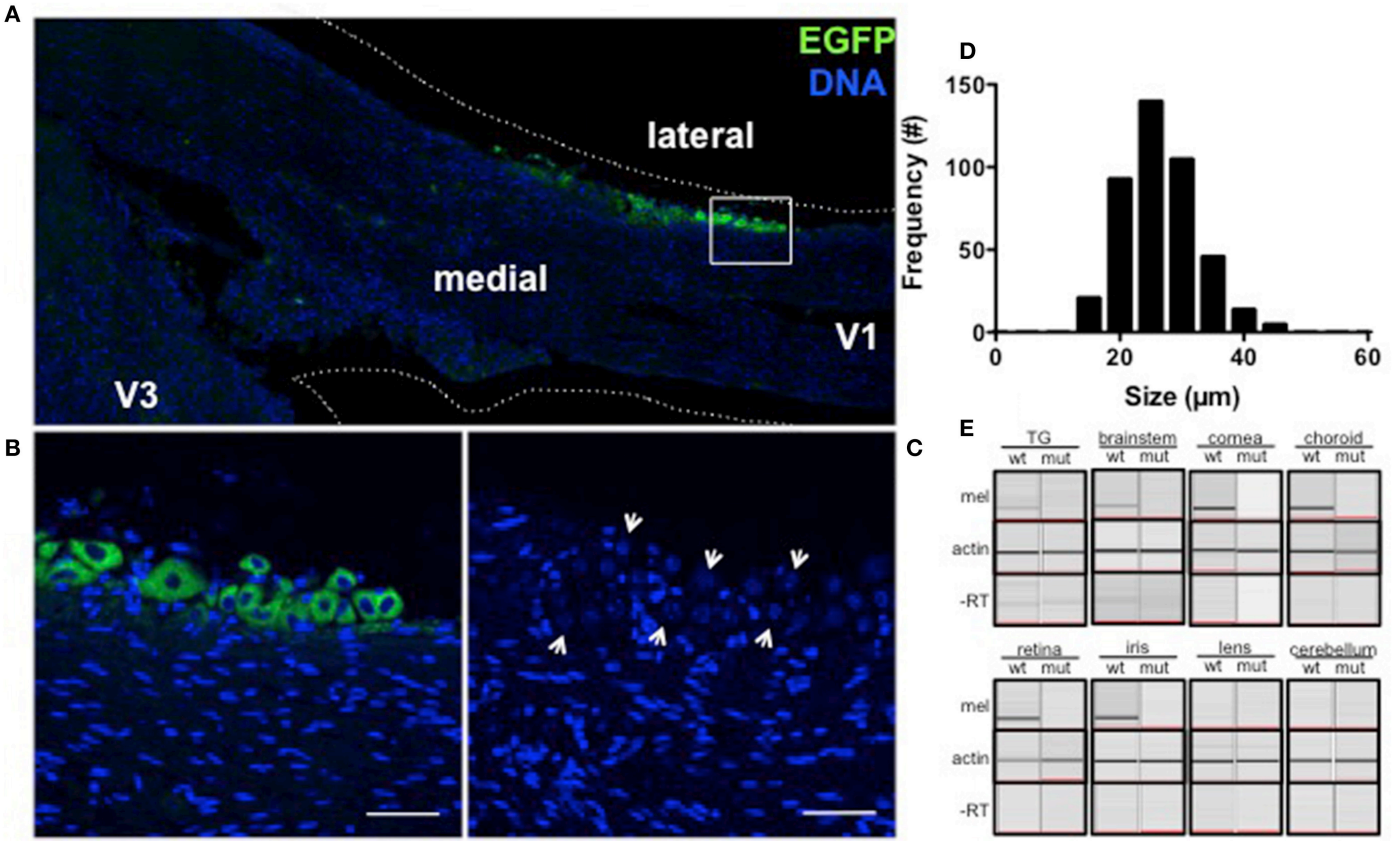
**Melanopsin is expressed in mouse TG neurons. (A)** EGFP (green) is expressed in a subset of trigeminal neurons localized to the V1 branch of OPN4^EGFP^ reporter mice. Nuclei are shown in blue. **(B)** EGFP-expressing TG neurons along the margin of the ganglia are shown at higher magnification (box from A). Scale bar = 50 μm. **(C)** A wild type littermate shows endogenous intrinsic cellular fluorescence in TG neurons (arrows) and no EGFP. Scale bar = 50 μm. **(D)** Frequency distribution of soma size shows EGFP-expressing TG neurons in OPN4^EGFP^ reporter mice range from 14 to 46 μm in diameter. **(E)** Melanopsin mRNA is expressed in the TG, brainstem, cornea, choroid (top), retina, and iris (bottom) of wild type mice but not OPN4^dta/dta^ mice (top) or in lens or cerebellum extracts (bottom) from wild type or OPN4^dta/dta^ mice. Actin is expressed in all samples (middle) and no PCR product is observed in the “no RT” controls (bottom).

The ability of light to elicit painful (Digre and Brennan, 2012) responses prompted us to investigate whether light detection could be mediated by melanopsin, a photopigment that underlies subconscious vision, in the trigeminal nerve. In fixed frozen sections of the TG of BAC transgenic melanopsin (OPN4^EGFP^) reporter mice (Figures 1A,B), a small percentage of TG neurons were EGFP fluorescent and easily distinguishable from other non-EGFP expressing TG neurons in the same sections and in neurons from wild type littermate controls (Figure 1C). Semi-quantitative morphometric analysis of bright EGFP-positive neurons in serial sections indicate melanopsin is expressed overall in approximately 3% (424/13191) of TG neurons: 85% (359/424) are small C fibers (14–30 μm), 15% (65/424) are medium Aδ fibers (30–50 μm) and 0% are large Aα or Aβ fibers (50–80 μm) neurons (Figure 1D, Le Pichon and Chesler, 2014). These neurons are distributed predominantly in lateral regions of the ganglia (403/424), with the majority in the V1 ophthalmic branch (256/424), but occasional EGFP-expressing neurons in the V2 maxillary (73/424), and the V3 mandibular (95/424) subdivisions, as observed in three ganglia from individual mice.

We analyzed tissue extracts from regions with peripherally and centrally targeted trigeminal axon projections, as well as the ganglia for melanopsin mRNA (Le Pichon and Chesler, 2014). Cornea, iris, choroid, brainstem (encompassing the trigeminal nuclei), and TG express melanopsin mRNA, but not lens, cerebellum or the same tissues from mice that express diphtheria toxin under control of the melanopsin promoter (OPN4^dta/dta^) (Matynia et al., 2012, 2015, Figure 1E). Melanopsin is deleted in this strain and the cells normally expressing melanopsin are ablated. Consistent with immunohistological reports (D. Copenhagen, personal communication), we detected melanopsin mRNA expression in corneal extracts that include the peripheral cornea with great care to avoid any pigmented tissue and the limbus but not in extracts from the central cornea (data not shown). Sequence analysis verified the exon-spanning amplified TG transcript is melanopsin (data not shown). This is the first report of melanopsin expression in the choroid, cornea, TG, and the brainstem.

**Figure 2:**
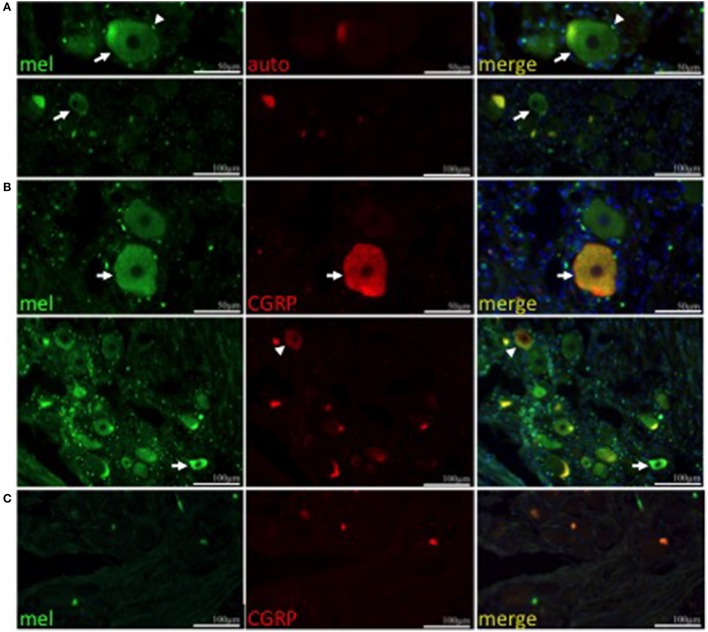
**Melanopsin is expressed in human TG neurons**. **(A)** Melanopsin (green) is expressed in human TG neurons (arrow) and satellite cells (arrowhead). Autofluorescence (red) is shown. **(A,B)** Nuclei are shown in blue. Upper panel, scale bar = 50 μm; lower panel, scale bar = 100 μm. **(B)** CGRP (red) colocalizes with melanopsin (green) in a population of neurons (top panel, arrow) however some neurons are immunopositive only for CGRP (lower panel, arrowhead) or melanopsin (lower panel, arrow). **(C)** Negative control using secondary antibodies. Scale bar = 100 μm.

To determine if trigeminal expression of melanopsin is conserved in humans, immunohistochemistry was performed on human trigeminal sections using an antibody specific to human and non-human primate melanopsin (Hannibal et al., 2004; Dacey et al., 2005; Gamlin et al., 2007). Trigeminal neurons showed cytoplasmic and plasma membrane immunolabeling (Figure 2A, upper and lower panel, respectively). Furthermore, numerous satellite glial cells were melanopsin positive, resulting in a perinuclear immunostaining, irrespective of whether or not the neuron they surround was melanopsin positive. Auto-fluorescence due to lipofuscin is present in the sections. We performed double immunostaining for calcitonin gene-related peptide (CGRP), a neuropeptide implicated in migraine (Eftekhari et al., 2015; Russo, 2015), and PACAP, which colocalizes with melanopsin in ipRGCs (Hannibal et al., 2004), to determine the identity of melanopsin-expressing TG neurons. Both melanopsin and CGRP are observed to co-localize in human trigeminal neurons (Figure 2B, top row). However, neurons that solely express melanopsin or CGRP are also observed (Figure 2B, bottom row). PACAP immunolabeling was very scarce and coupled with the few melanopsin labeled cells, we were unable to observe neurons with co-localization of these two markers (data not shown). Immunostaining was specific to the anti-melanopsin primary antibody (Figure 2C).

### Melanopsin expressing trigeminal neurons are intrinsically photosensitive

**Figure 3:**
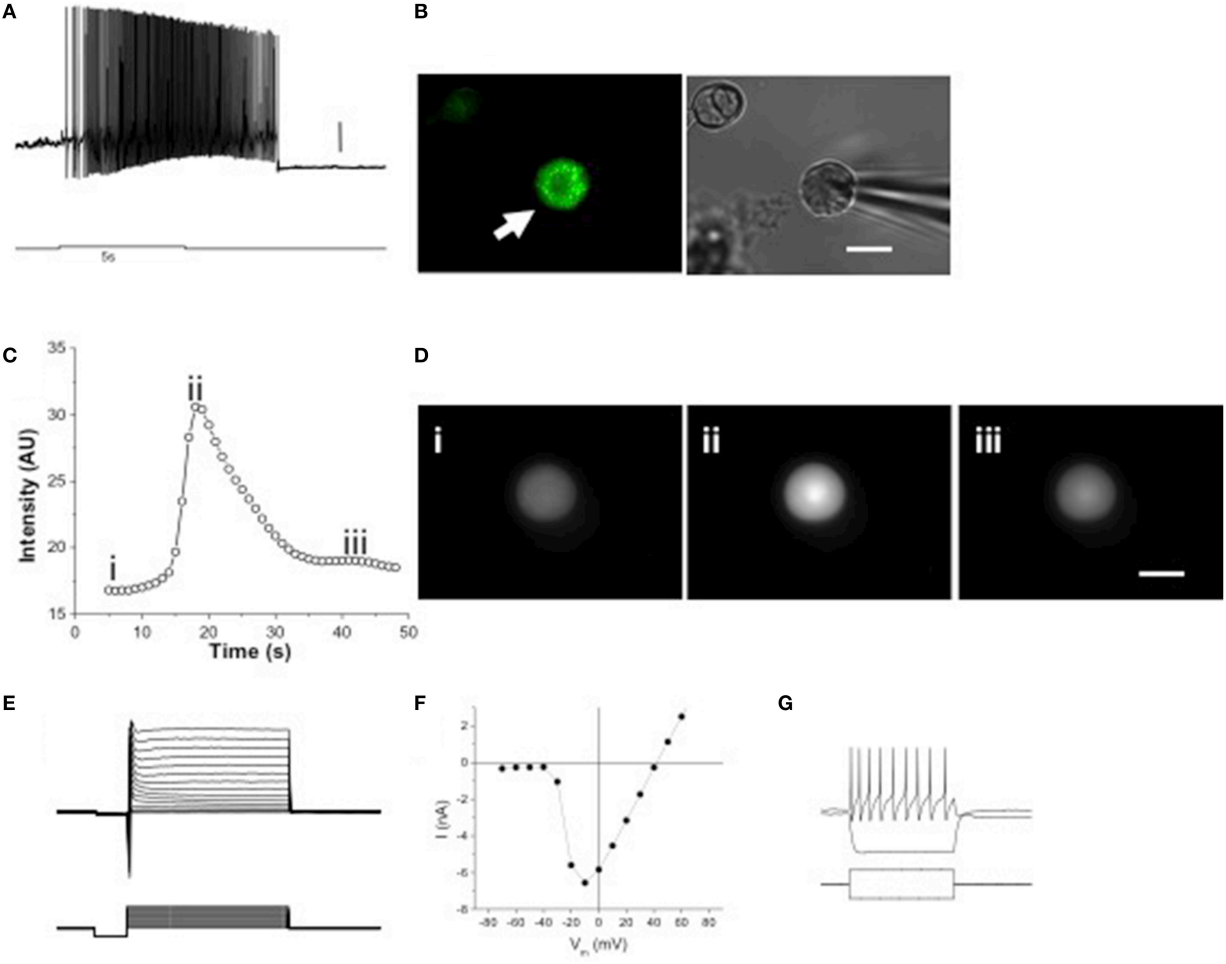
**Melanopsin expression confers intrinsic photosensitivity to TG neurons. (A)** 5 s 480 nm stimulus initiated delayed onset firing of action potentials in current clamp recordings. Scale bar = 10 mV. **(B)** Dissociated OPN4 trigeminal neurons were identified by EGFP fluorescence (left panel, arrow). Trigeminal neurons not expressing EGFP were identified by brightfield illumination (right panel, arrowhead). Scale bar = 20 μm. **(C)** Constant 480 nm light stimulated slow, inactivating calcium signals in some isolated TG neurons from wild type mice, measured with fluo-4 AM. **(D)** Freeze frame [Ca^2+^]_i_ images taken before stimulation (i), at the peak of the Ca^2+^ signal (ii) and after return to normal (iii). Scale bar = 20 μm. **(E)** Voltage clamp recording showing Na^+^ and K^+^ currents in response to voltage clamp steps of 100 ms duration. **(F)** Current-voltage relation of isolated TG neuron shown in *E*. **(G)** Current clamp recording of an isolated TG neuron showing response to 10 pA hyperpolarizing and depolarizing current injections, the latter producing a train of action potentials. The current injection steps lasted 100 ms.

To determine if melanopsin expression confers intrinsic photosensitivity to these cells, we performed patch clamp recordings and calcium imaging of acutely dissociated, dark-adapted OPN4^EGFP^ or wild type TG neurons, respectively. Stimulation with bright, blue light (6.8 × 10^8^ photons/s/μm^2^; 480 nm, a light level equivalent to noon light levels on a clear sunny day; Enezi et al., 2011) caused a volley of action potentials with a characteristic delay after stimulus onset and sustained firing after light stimulation ends, as shown in a whole cell patch clamp recording of a dissociated EGFP-positive TG neuron (Figures 3A,B). ipRGCs exhibit a similar delayed onset and sustained firing of action potentials (Hartwick et al., 2007; Schmidt and Kofuji, 2009). A second silent state was observed after stimulus offset and cessation of action potentials, which has recently been observed in ipRGCs (Emanuel and Do, 2015). For calcium imaging experiments, dissociated neurons from wild type mice were used to avoid interference from EGFP, which has similar excitation and emission wavelengths as fluo-4 (Paredes et al., 2008). Calcium imaging revealed that intracellular calcium increased in response to light stimulation in 3 out of 93 fluo-4 loaded isolated wild type TG neurons (Figures 3C,D). Dissociated TG neurons had peak Na^+^ currents of 6.21 ± 0.87 nA (*n* = 23) at −10 mV under voltage clamp (Figures 3E,F) and sustained spiking was observed with injected current (Figure 3G). These results show that melanopsin-expressing TG neurons have intrinsic photosensitivity.

### A potential alternative *in vivo* circuit for light detection

**Figure 4:**
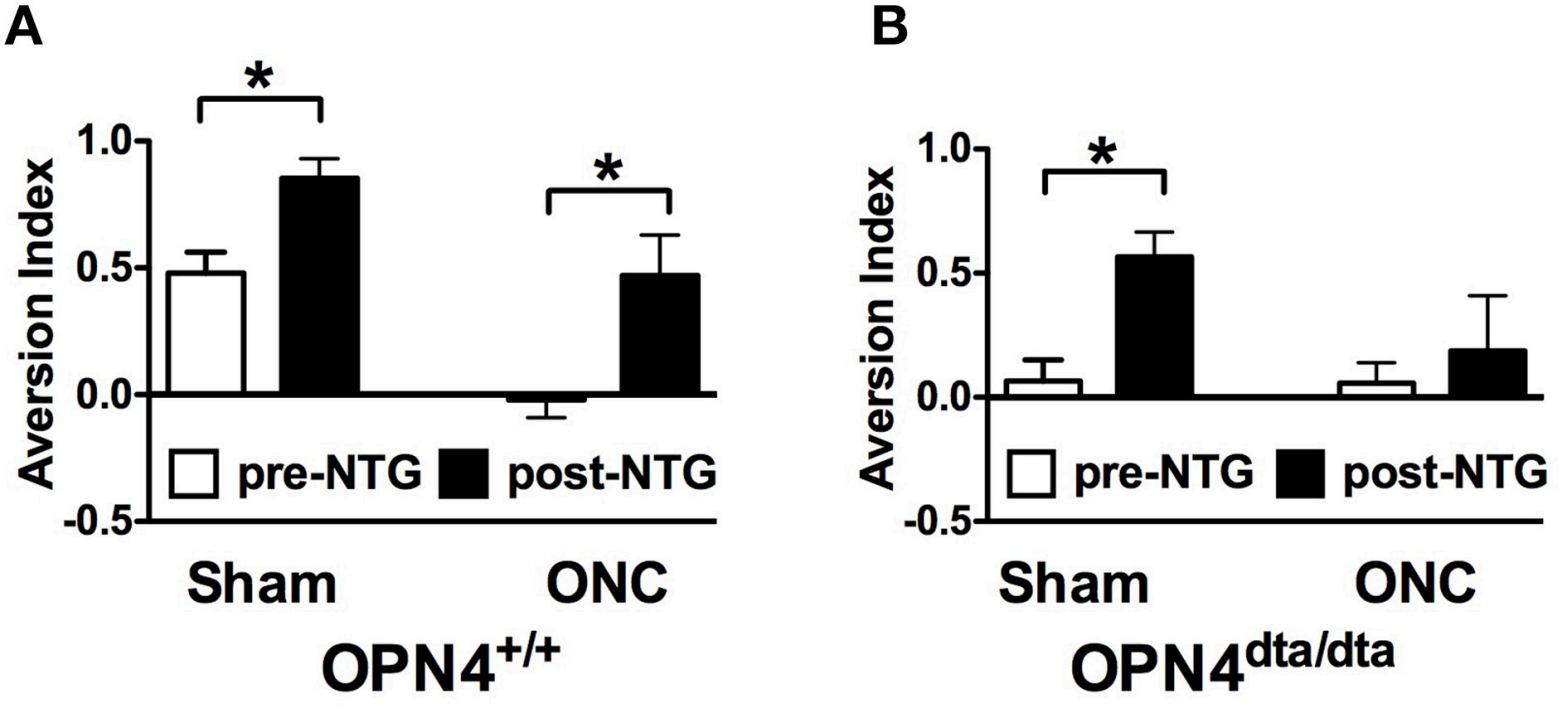
**Latent light detection after ONC is revealed by administration of NTG**. Light aversion was tested pre-injection (white) and post-injection (black) of 10 mg/kg NTG. **(A)** OPN4^+/+^ wild type mice with sham surgery (*n* = 8, *T*_14_ = 3.3, *p* = 0.005) and ONC (*n* = 8, *T*_14_ = 2.8, *p* = 0.01) show increased light aversion with NTG. **(B)** OPN4^dta/dta^ mice with sham surgery (*n* = 8, *T*_14_ = 3.8, *p* = 0.002) show increased light aversion with NTG whereas OPN4^dta/dta^ mice with ONC (*n* = 8, *T*_14_ = 0.5, *p* = 0.6) show no increase in light aversion. Data are represented as mean ± SEM, Students *t*-test and *p*-values are given. ^*^ *p* < 0.05.

With the ability to respond to light *ex vivo*, melanopsin-expressing TG neurons were tested for the potential to detect light *in vivo*. To differentiate light responses of TG neurons from ipRGCs, mice with severe bilateral optic nerve crush (ONC) or sham operated (sham) were tested 1 month after surgery when the majority of RGCs have degenerated (Perez de Sevilla Muller et al., 2014). In mice, consequences of optic nerve transection include transecting multiple trigeminal nerves in and near the meningeal sheath and transecting the ophthalmic artery that results in loss of trigeminal innervation and retinal blood flow, and ultimately retinal death and significant inflammation (Levkovitch-Verbin, 2004). To spare damage to the trigeminal nerves, we performed ONC. Since mice with optic nerve damage or lacking ipRGCs have deficits in pupil constriction, all light aversion experiments were performed in mice with pharmacologically fully dilated pupils. ONC mice exhibited no light aversion to 1000 Lux illumination, acting “blind” as expected (Figure 4A). To test light-dependent behavioral responses in a pathophysiological condition, nitroglycerin (NTG) injection was used as a model of migraine. In addition to sensitizing the trigeminal ganglia, NTG causes immediate vasodilation, but 1 h after injection, vascular caliber has returned to pre-injection levels while pain and trigeminal responses are amplified (Olesen et al., 1994; Tassorelli et al., 2005; Schoonman et al., 2008; Pradhan et al., 2014). ONC mice exhibited strong light aversion 60 min after nitroglycerin (NTG; Figure 4A). By contrast, light aversion was significantly reduced, showing no difference pre- and post-injection in OPN4^dta/dta^ mice lacking melanopsin-expressing neurons with the same severe bilateral ONC (Figure 4B). Sham for both wild type and OPN4^dta/dta^ mice showed enhanced light aversion with NTG (Figures 4A,B).

**Figure 5:**
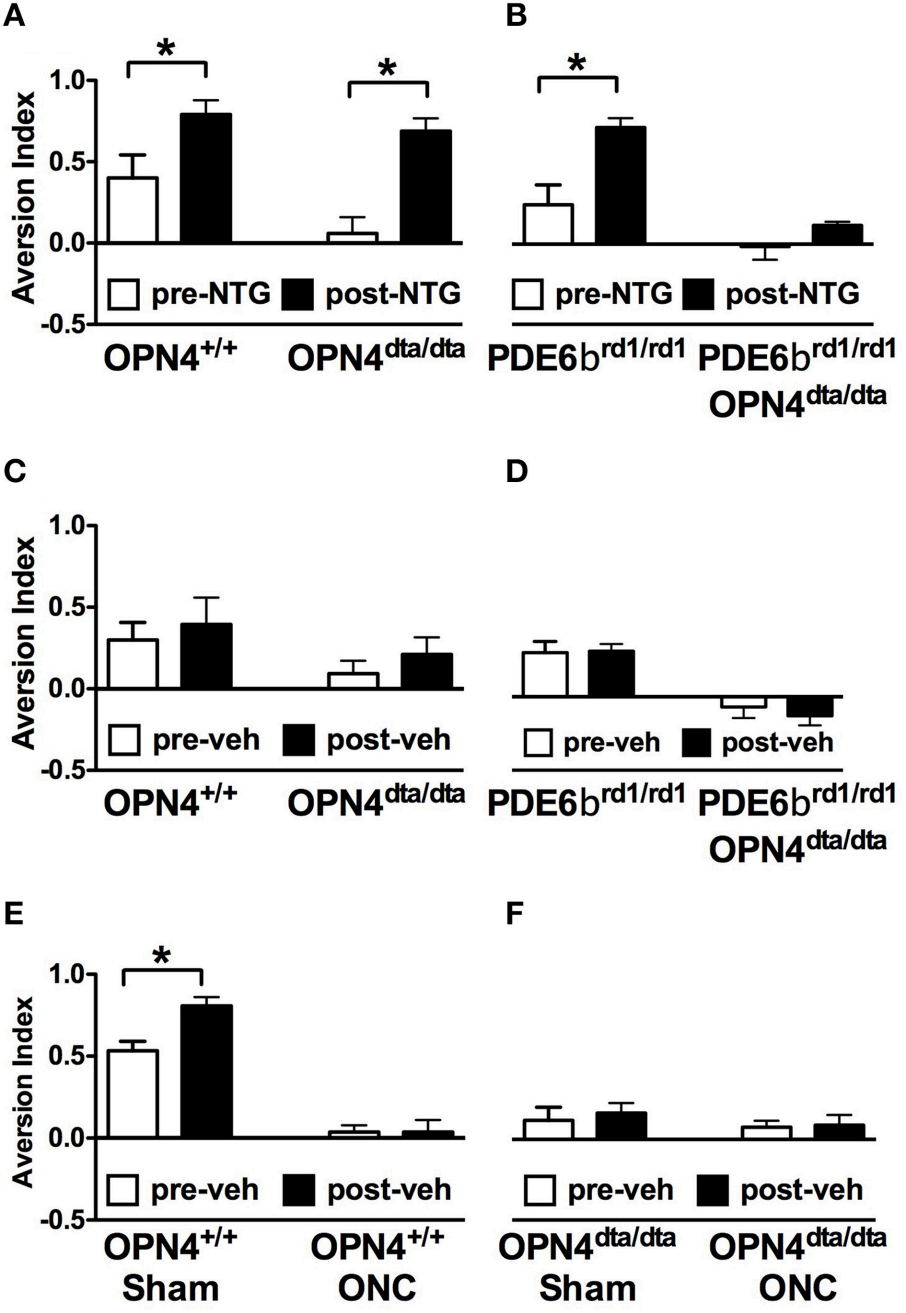
**Light aversion is enhanced by NTG and requires rods, cones and melanopsin-expressing neurons**. Light aversion was tested pre- (white) and post-injection (black) of 10 mg/kg NTG in **(A)** OPN4^+/+^ wild type mice (*n* = 8, *T*_14_ = 2.4, *p* = 0.03) or OPN4^dta/dta^ mice lacking melanopsin-expressing neurons (*n* = 9, *T*_16_ = 5.0, *p* = 0.0001) and **(B)** PDE6β^rd1/rd1^ mice lacking rod and cone photoreceptors (*n* = 9, *T*_16_ = 3.5, *p* = 0.003) or PDE6β^rd1/rd1^;OPN4^dta/dta^ mice lacking rods, cones and melanopsin-expressing neurons (*n* = 4). Light aversion pre- (white) and post-injection (black) of vehicle is shown for **(C)** OPN4^+/+^ wild type (*n* = 9) or OPN4^dta/dta^ mice (*n* = 9); **(D)** PDE6β^rd1/rd1^ mice (*n* = 9) or PDE6β^rd1/rd1^;OPN4^dta/dta^ mice (*n* = 4); **(E)** OPN4^+/+^ wild type with sham surgery (*n* = 8, *T*_14_ = 3.4, *p* = 0.004) or ONC (*n* = 8); and **(F)**; OPN4^dta/dta^ mutant mice with sham surgery (*n* = 5) or ONC (*n* = 5). Data are represented as mean ± SEM, students *t*-test and *p*-values are given. ^*^ *p* < 0.05.

NTG increased light aversion in wild type mice, OPN4^dta/dta^ mice, and PDE6β^rd1/rd1^ mice with degeneration of rod and cone photoreceptors, but no increase in light aversion was observed after NTG injection in the double mutant OPN4^dta/dta^;PDEβ β^rd1/rd1^ mice (Figures 5A,B). Light aversion is not affected by vehicle injection in any group except wild type mice with sham surgery, most likely a non-specific effect (Figures 5C–F). Nitric oxide donors similar to NTG increase retinal responses to light *during* drug application but the duration is unknown (Vielma et al., 2010). Illumination-response curves for full field electroretinography (ERG) performed 60 min post-injection (10 mg/kg NTG) in wild type mice show *a*-wave and *b*-wave maximal responses and sensitivity are the same as vehicle-injected controls (Table 1).

**Table 1:** **Retinal electrophysiological responses to NTG**.

***a*****-waves**						
	**V_max_ (μV)**	**Sem**	***n***	**K_m_ (cd–s/m^2^)**	**Sem**	***n***
**SCOTOPIC**						
NTG	−343	17	6	0.038	0.003	6
Vehicle	−381	33	5	0.048	0.003	5
***b*****-waves**						
**SCOTOPIC**						
NTG	−554	35	6	0.156	0.27	6
Vehicle	−599	56	5	0.127	0.011	5
**PHOTOPIC**						
NTG	214	17	6			
Vehicle	204	16	5			

**Figure 6:**
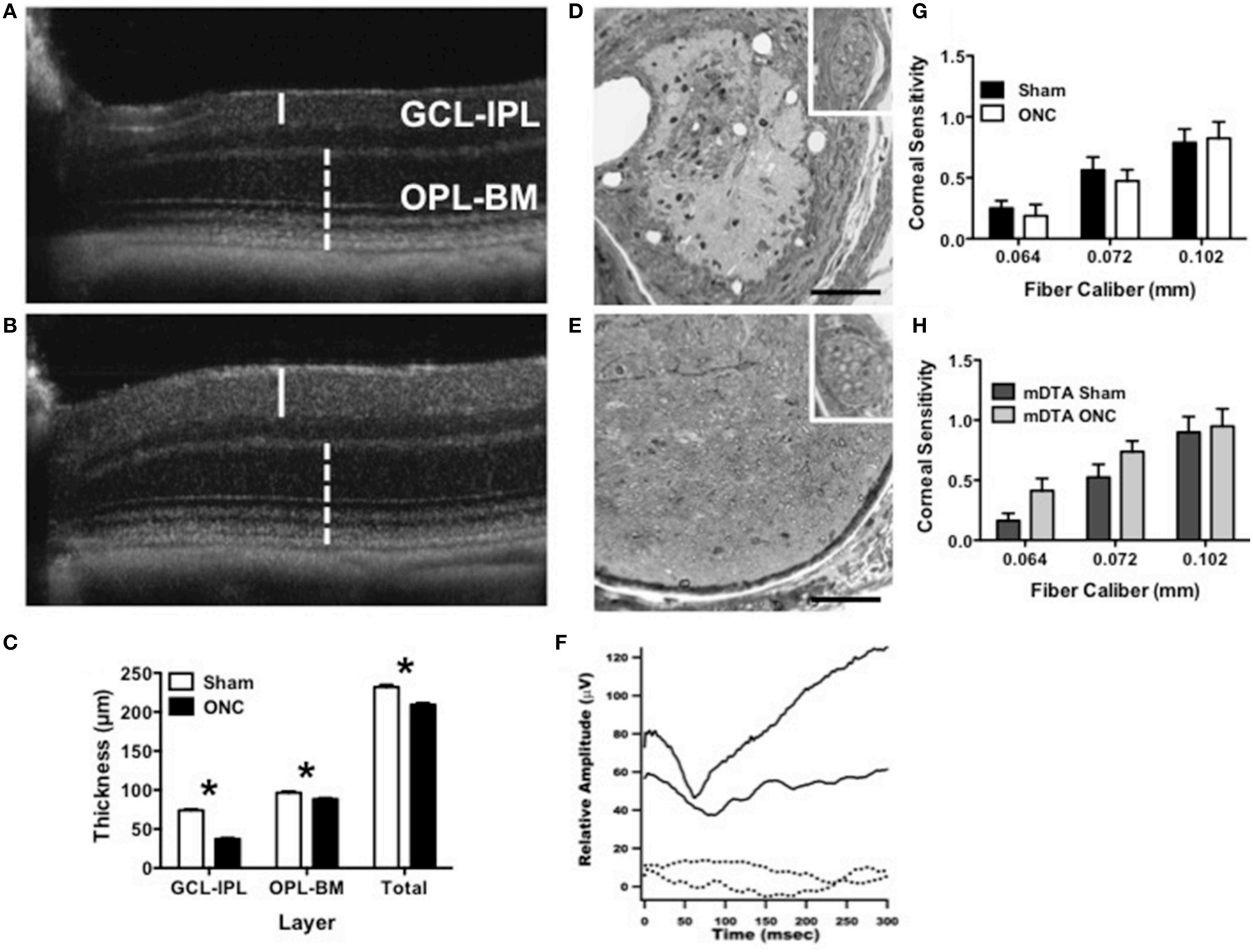
**ONC causes degeneration of RGCs and the optic nerve with no alteration to corneal innervation**. **(A–C)** Degeneration of the ganglion cell layer 45 days after ONC was assessed using *in vivo* sd-OCT imaging. Representative images are shown for **(A)** ONC and **(B)** sham. The inner retina containing retinal ganglion cells (GCL-IPL, solid line) showed greater degeneration than the outer retina containing rod and cone photoreceptors (OPL-BM, dashed line). **(C)** The average thickness for sham and ONC is shown for the GCL-IPL (*T*_14_ = 21.4, *p* < 0.0001), OPL-BM (*T*_14_ = 3.6, *p* = 0.003) and the total retinal thickness (Total, *T*_14_ = 6.1, *p* < 0.0001). **(D,E)** Optic nerve damage was assessed by light microscopy with representative cross-sectional images approximately 0.5 mm from the globe shown for **(D)** ONC and **(E)** sham. Inset shows intact trigeminal nerve bundles. Scale bar = 100 μm. **(F)** OPN4^dta/dta^ mice with sham (solid line) showed normal P1-N1 response amplitudes whereas the VEP was not recordable in OPN4^dta/dta^ mice with ONC (dashed line). **(G,H)** Trigeminal innervation of corneal mechanosensitivity was tested using von Frey fibers (0.064–0.102 mm diameter) in **(G)** OPN4^+/+^ mice with sham (black, *n* = 16 eyes) or ONC (white, *n* = 16 eyes) and **(H)** OPN4^dta/dta^ mice with sham (black, *n* = 16 eyes) or ONC (gray, *n* = 16 eyes), showing the same corneal sensitivity (no interaction of surgery by fiber caliber with no effect of surgery but a main effect of fiber caliber [*F*_(2, 90)_ = 17, *p* < 0.0001, *F*_(2, 78)_ = 14, *p* < 0.0001, respectively]). Data are represented as mean ± SEM, students *t*-test and *p*-values are given. ^*^ *p* < 0.05.

Injury was specific to the optic nerve with minimal-to-no collateral damage (Figure 6). Degeneration of the ganglion cell layer (GCL-IPL) was confirmed by sd-OCT imaging (Figures 6A–C, Perez de Sevilla Muller et al., 2014). Retinal light responses were normal 5 weeks after surgery for both photopic *a*-waves (Sham: −220 μV ± 20, *n* = 3; ONC: −163 μV ± 14, *n* = 5; *T*_6_ = 2.3, *p* = 0.06) and *b*-waves (Sham: 406 μV ± 79, *n* = 3; ONC: 367 μV ± 46, *n* = 5; (*T*_6_ = 0.46, *p* = 0.66) as assessed by electroretinography at 0.05345 cd/m^2^. No myelinated axons were detectable in the optic nerve (Figures 6D,E). Consistently, cortical light responses were undetectable in ONC mice 60 min after NTG injection using VEPs (Figure 6F). Pupil constriction in response to light was either absent (*n* = 6) or significantly reduced (*n* = 2) in ONC mice (2.75 ± 0.16 AU) compared to normal in sham (1.00 ± 0.00). Trigeminal nerve bundles that run parallel to the optic nerve remained intact after ONC (inset, Figures 6D,E). Furthermore, trigeminal nerve function was unaffected in either wild type or OPN4^dta/dta^ mice assessed by corneal mechanical sensitivity test (Figures 6G,H, respectively).

## Discussion

Photoreceptors, pinealocytes in non-mammalian vertebrates, deep brain photoreceptors in fish, eels and birds, melanophores, and mechanoreceptors in frogs, and melanocytes in humans all express active opsins ranging from rhodopsin to pinopsin to melanopsin that mediate light-dependent processes (Blackshaw and Snyder, 1997; Provencio et al., 1998b; Kojima and Fukada, 1999; Peirson et al., 2009; Wicks et al., 2011; Baker et al., 2015). Among opsins, melanopsin is attributed with the broadest range of functions, having recently been shown to influence contrast vision, vasorelaxation, and vascular development in addition to its established roles in circadian photoentrainment, PLR, light aversion, and negative masking (Berson et al., 2002; Hattar et al., 2002; Panda et al., 2002; Gooley et al., 2003; Lucas et al., 2003; Schmidt et al., 2014). Here, we describe the discovery of melanopsin expression in classic pain sensory neurons that renders them photosensitive.

### TG neurons express melanopsin and are intrinsically photosensitive

We showed that a small subset of neurons express melanopsin in both human and mouse TG tissue. In mice, we used a cytoplasmic EGFP reporter to identify melanopsin, a 7-transmembrane protein that localizes to the plasma membrane (Hattar et al., 2002; Qiu et al., 2005). In human TG tissue, melanopsin was localized to the cytoplasm and plasma membrane. The observed cytoplasmic localization of melanopsin may be a result of the time post-mortem of tissue procurement as similar staining has been observed in human retina with this antibody (Hannibal et al., 2004; La Morgia et al., 2011). Although melanopsin has been reported in other retinal neurons (Dkhissi-Benyahya et al., 2006), the perinuclear staining of trigeminal glial satellite cells is unexpected and requires further investigation beyond the scope of this report.

With more than 13 mammalian opsin family members, many with no identified biological activity, expression of opsin alone is insufficient to endow photosensitivity on a cell. We therefore showed that isolated neurons, devoid of neural, systemic, or local control or feedback, fire a volley of action potentials and mobilize calcium in response to blue light. Intrinsic responses of both ipRGCs and TG neurons showed delayed onset and sustained firing in response to blue light. ipRGCs were recently shown to have tristability with two silent states and one signaling state that may allow light-independent regeneration of chromophore (Emanuel and Do, 2015). To determine if melanopsin-expressing TG neurons also have three states, as suggested by different activity pre- and post-firing, further experiments would be needed. These results provide the necessary evidence that the molecular machinery for melanopsin-mediated light-driven responses is functional in this select class of TG neurons, and provide crucial proof-of-concept for biological relevance of this system.

### Comparison of melanopsin-expressing TG neurons and ipRGC

Other than melanopsin and its ability to respond to light, ipRGCs and TG neurons that express melanopsin share very few characteristics. Different classes of ipRGCs have overlapping somatic sizes from 12 to 26 μm (Hattar et al., 2002), the largest of which are M4 ipRGCs, and are classified on their dendritic arborization and intrinsic electrophysiological light responses that are reflective of melanopsin levels (Schmidt et al., 2011; Estevez et al., 2012). Melanopsin-expressing TG neurons range from 14 to 46 μm, classifying them as C fiber or Aδ fiber neurons (Le Pichon and Chesler, 2014). TG neurons are pseudounipolar neurons that lack dendrites but co-express specific genes that respond to distinct stimuli. Predominant localization of melanopsin-expressing TG neurons on the lateral margin suggests that melanopsin will be co-expressed in multiple different classes since each class (e.g., transient receptor potential channel melastatin 8, TRPM8) tends to be distributed throughout the ganglion (Knowlton et al., 2013).

ipRGCs have both intrinsic and extrinsic light responses from melanopsin, and rod and cone photoreceptors via their inner retinal circuits, respectively (Schmidt et al., 2011). By contrast, TG neurons do not receive sensory input from other neurons but instead expresses a single or small number of receptors that define their sensory profile (Le Pichon and Chesler, 2014). In the retina, melanopsin colocalizes with PACAP (Hannibal et al., 2002, 2004, 2014; Hannibal and Fahrenkrug, 2004), however, we were unable to determine this relationship in human TG due to the low number of neurons labeled by both antibodies. Instead, we determined that melanopsin co-localizes with CGRP, a neuropeptide known to be expressed in small and medium (C fiber and Aδ fiber) TG neurons that modulate vascular caliber and nociception, with a role in migraine pathophysiology (Edvinsson et al., 1987; Felipe et al., 1999; Tajti et al., 1999; Hou et al., 2001; Eftekhari et al., 2010; Russo, 2015). Melanopsin-expressing TG neurons also likely express receptors for other sensory stimuli, for example, TRPM8 for temperature and evaporation in small (10–30 μm) C fibers or piezo2 for pressure in medium-sized (30–50 μm) Aδ fibers (Robbins et al., 2012; Bron et al., 2014; Le Pichon and Chesler, 2014). We previously identified a decrease in corneal mechanical sensitivity in OPN4^dta/dta^ mice, consistent with ablation of melanopsin-expressing mechanoreceptive Aδ fibers (Matynia et al., 2015). Coincident stimulation of melanopsin-expressing TG neurons with their cognate ligand and light may lead to enhanced responses to normal and noxious stimuli.

### Melanopsin activation *in vivo* in TG neurons

For trigeminal neurons to be activated by light *in vivo*, sufficient levels of melanopsin must be present in sites accessible to light. Melanopsin expression was found in sites containing both trigeminal somata and projections (cornea, choroid, iris), suggesting protein may also be expressed in axons. From the somata, we estimated melanopsin expression in TG neurons to be comparable to the lower-expressing, non-M1, non-M2 ipRGCs in mouse retinas since physiological responses required stronger light stimulation compared to M1 and M2 but similar to M3-M5 ipRGCs. For isolated melanopsin-expressing trigeminal neurons, we used 5 s of 480 nm light at 6.8 × 10^16^ photons/s/cm^2^ compared to stimulation ranging from 3.25 × 10^12^ photons/s/cm^2^ for 30 s of white light to 1.2 × 10^17^ photons/s/cm^2^ for 10 s of 480 nm light in other studies of M1-M5 ipRGCs (Schmidt and Kofuji, 2009; Ecker et al., 2010; Estevez et al., 2012; Hu et al., 2013; Sodhi and Hartwick, 2014). By contrast, mouse M1 ipRGCs are ~10^6^ times less sensitive than rod photoreceptors and 10^4^ times less sensitive than cone photoreceptors (Do et al., 2009). Consistently, EGFP fluorescence was lower in TG neurons compared to M1 and M2 ipRGCs. This low melanopsin level may similarly ensure a low probability of photon capture (Do et al., 2009) and ensure only bright, potentially harmful blue light activates TG neurons. These characteristics are consistent with light-induced effects on nociception that are experienced in every individual by sufficiently strong light, and by dimmer light in individuals with photoallodynia (Digre and Brennan, 2012).

**Figure 7:**
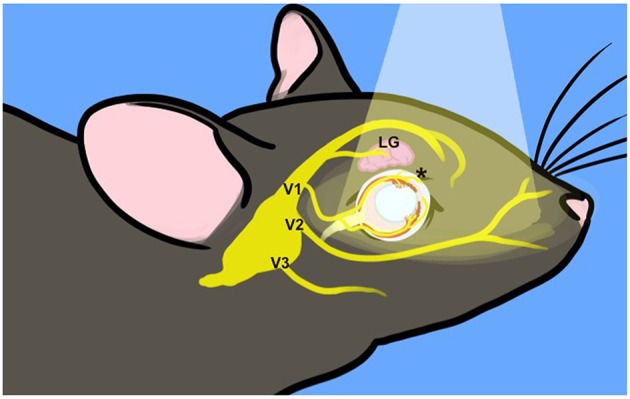
**Incident light reaches trigeminal nerve terminals**. Surface structures innervated by the trigeminal nerve are exposed to incident light, indicated by the pale cone. The TG are situated at the base of the skull. Three major branches exit the skull to target ocular (V1, ophthalmic), upper jaw (V2, mandibular), and lower jaw (maxillary) structures. The frontal branch of V1 innervates the lacrimal gland (LG) and forehead. The ciliary branch innervates the cornea, iris and choroid (^*^) as well as the nasal mucosa (not depicted). For illustration purposes, many trigeminal branches are not shown and the depiction is not to scale.

Clustering of melanopsin-expressing TG neurons on the lateral margin suggests that they innervate a variety of targets (Felipe et al., 1999). TG somata are located within the skull at the base of the brain and any light that penetrates to the cell bodies of trigeminal neurons would be significantly attenuated. However, their dendrites extend into the superficial layers of the cornea, the first point of contact for light entering the eye as well as the iris, choroid, and lacrimal glands (Figure 7). Depolarization resulting from light stimulation of melanopsin at any of these peripheral sites and its transduction pathway in these small nerve endings could initiate action potentials that are conducted into the trigeminal ganglion. Light (550 nm and below) is attenuated at the retina by up to 80% in adults (Hecht et al., 1942; Kessel et al., 2010), suggesting that small amounts of melanopsin in the cornea, iris choroid or other peripheral tissue might be sufficient for *in vivo* melanopsin photoactivation (Do et al., 2009).

### Optic nerve function is completely disrupted by severe bilateral ONC

The presence of photo-responsiveness in rod/cone-blind individuals spurred the search for a non-rod, non-cone photoreceptor, leading to the discovery of melanopsin (Provencio et al., 1998a). We mimicked this approach, using a severe bilateral ONC injury since manipulation of melanopsin-expressing neurons would equally affect retinal and TG neurons. Histologically, the optic nerve had no detectable axons but the ciliary V1 trigeminal nerve bundles remained intact. Functionally, ONC mice with no further manipulation acted “blind” in a behavioral light aversion task and had no PLR even though the retina responded normally to light and corneal sensitivity was normal. Therefore, within the sensitivity of available tests, the optic nerve was non-functional however, it cannot be absolutely ruled out that a minimal number of unmyelinated fibers were intact. Optic nerve transection would ensure complete loss of optic nerve transmission but would also sever multiple trigeminal nerve branches and cause substantial pathology from transection of the ophthalmic artery. Previously, innate light aversion was shown to be dependent on melanopsin-expressing neurons. With the requirement for a functional optic nerve, ipRGCs most likely mediate innate light aversion while light hypersensitivity associated with pathophysiological states may use the same or recruit additional neural systems (Johnson et al., 2010; Thompson et al., 2010; Matynia et al., 2012, 2015).

### Melanopsin-expressing TG neurons as a potential *in vivo* light detecting circuit

In this study, NTG induced light aversion at non-aversive, moderate illumination levels at a dose and time that is known to activate TG neurons, increase pain responses, and trigger migraine in susceptible individuals and migraine- and pain-related symptoms in mice (Olesen et al., 1994; Tassorelli et al., 2005; Pradhan, 2012). The difference in light aversion between pre- and post-NTG is comparable to the difference in light aversion between 0 and 1000 lux in mice with dilated pupils (Matynia et al., 2012). While there is not a linear relationship between light aversion and light levels, this is nonetheless indicative of a high degree of sensitization. Although melanopsin is expressed in the iris and ciliary marginal zone, which contribute to the PLR and could stimulate trigeminal nerves (Xue et al., 2011; Semo et al., 2014), pupil constriction can be excluded as a mechanism for optic nerve-independent, nitroglycerin-induced light aversion as all mice were fully dilated pharmacologically. Two pieces of evidence support a role for melanopsin-expressing TG neurons in NTG-induced light aversion. First, at the same time after NTG administration when vascular caliber has returned to normal and tactile and thermal hypersensitivity are observed (Bates et al., 2010; Pradhan et al., 2014), we observed severe light aversion in ONC mice with no concomitant increase in retinal responses to light and no detectable cortical responses to light (Olesen et al., 1994; Tassorelli et al., 2005; Schoonman et al., 2008; Pradhan et al., 2014). Second, this ONC-independent NTG-induced light aversion required the presence of melanopsin-expressing neurons. Ablation of melanopsin-expressing neurons was verified before behavioral testing by loss of PLR, which is mediated by M1 ipRGCs that express the highest level of melanopsin (Xue et al., 2011). By contrast, lower expressing neurons may not be completely ablated and may contribute to phenotypic variability (Guler et al., 2008). Melanopsin expression has also been demonstrated in vascular tissue but until the cellular identity of this melanopsin source is elucidated, any potential connection with the trigeminovascular system, which mediates vasodilation, remains speculative (Sikka et al., 2014).

### Clinical and functional implications

The discovery of melanopsin expression in TG neurons provides direct evidence for a novel functional neural circuit originating in the peripheral nervous system whereby light likely influences multiple sensory processes. In specific pathophysiological conditions that emerge in disease, thresholds for activation of C fiber or Aδ trigeminal neurons may be lowered (Latremoliere and Woolf, 2009), increasing the likelihood of the contribution of melanopsin-mediated responses to symptoms. For example, sensitization of TG neurons in models (NTG) or clinical conditions may lead to photoallodynia, a significant comorbidity in migraine, corneal surface damage, and many other injuries or disease (Digre and Brennan, 2012), many of which affect significant populations worldwide (Lipton et al., 2007; Gayton, 2009). CGRP is currently being developed as a target molecule for pharmacological treatment of migraine (Russo, 2015). Colocalization of melanopsin and CGRP in human TG neurons suggests these new drugs may effectively reduce migraine-related photophobia, which currently has no treatment other than light avoidance. Other physiological functions that may be influenced by trigeminal melanopsin include ACHOO Syndrome (autosomal dominant compelling helioophthalmic outburst), a genetic condition in which bright sunlight triggers sneezing in about 25% of the population (Garcia-Moreno, 2006), the ability of rod/cone blind patients to perceive light (Amini et al., 2006; Noseda et al., 2010; Vandewalle et al., 2013), the use of phototherapy to treat seasonal affective disorder and non-seasonal depression (Golden et al., 2005); and color sensation synesthesia, with cross-talk between pain and color (Novich et al., 2011).

In summary, we have identified an active photopigment, melanopsin, in the classic trigeminal pain and mechanosensitivity circuitry that provides a new mechanistic insight to light detection in the CNS via a non-optic nerve pathway with applications to both basic and clinical sciences. Defining the neural pathways that convey irradiance information pertinent to light-induced pain has important implications for clinical treatment of intractable photoallodynia and will give a better comprehension of the interaction between visual and trigeminal sensory processes.

## Author contributions

AM conceived and designed experiments. LE, SB, NB, AC, and MG gave advice on experimental design. AM, XS, EN, FWB, SP, SB, LP, JK, ZW, AR, SH, PK, and AW performed and analyzed experiments. LP, SN, SB, AC, NB, and MG gave advice on data analysis. AM wrote the paper.

## Funding

This work was supported in part by the Department of Defense (PR100085) and the NEI (EY00331 and EY04067). We also gratefully acknowledge financial support by an unrestricted grant from Research to Prevent Blindness, Inc., to the Department of Ophthalmology; the Harold and Pauline Price Chair in Ophthalmology, the Gerald Oppenheimer Family Foundation Award for the Prevention of Eye Disease, and the Jules Stein Eye Institute to MG; the Knights Templar Eye Foundation to AM; and the Plum Foundation to SB and NB. NB is a VA Career Research Scientist.

### Conflict of interest statement

The authors declare that the research was conducted in the absence of any commercial or financial relationships that could be construed as a potential conflict of interest.
